# 2-Mercaptobenzimidazole Functionalized Copper Nanoparticles Fluorescence Probe for Sensitivity and Selectivity Detection of Cys in Serum

**DOI:** 10.3390/s23135814

**Published:** 2023-06-22

**Authors:** Jing Liu, Xiaozong Dou, Hongyan Zhang

**Affiliations:** 1 Xinjiang Key Laboratory of Solid State Physics and Devices, Xinjiang University, Urumqi 830017, China; xdlj@xju.edu.cn (J.L.); dxz20dyjy@163.com (X.D.); 2 School of Physical Science and Technology, Xinjiang University, Urumqi 830017, China

**Keywords:** fluorescent probe, monovalent copper, metal-ligand charge transfer transition, cysteine detection, serum

## Abstract

In this paper, a 2-mercaptobenzimidazole-copper nanoparticles (MBI-CuNPs) fluorescent probe with high performance based on 2-mercaptobenzimidazole functionalized copper nanoparticles was synthesized by a hydrothermal method and used for cysteine (Cys) detection in serum. The MBI-CuNPs probe exhibits strong fluorescence emission at 415 nm under the excitation at 200 nm, which is attributed to the metal-ligand charge transfer (MLCT) transition through the coordination of an MBI ligand and monovalent copper. Furthermore, the MBI-CuNPs probe has a high quenching fluorescence response to Cys, and shows a good linearity relationship with Cys in 0.05–65 µM, with a detection limit of 52 nM. Moreover, the MBI-CuNPs probe could eliminate the interference of biological mercaptan Hcy and GSH with a similar structure and reaction properties, due to the strong electron-donating ability of Cys, which can quench the fluorescence of the MBI-CuNPs probe. The MBI-CuNPs probe was applied to the analysis of Cys in real serum, and the absolute recovery rate was as high as 90.23–97.00%. Such a fluorescent probe with high sensitivity and selectivity has potential applications for the early prevention of various diseases caused by abnormal Cys levels.

## 1. Introduction

Cysteine (Cys), homocysteine (Hcy) and glutathione (GSH) are the main mercaptans existing in organisms and maintain the normal physiological functions and metabolism of organisms. Abnormal levels of biological thiols will hinder the normal physiological function of organisms and cause various diseases [1,2]. In the biological thiols, the content of Cys in the blood has an important effect on human health. For example, Cys deficiency will cause muscle atrophy, hematopoiesis reduction, edema, liver damage, white blood cell loss, skin lesions, hair loss, lethargy and psoriasis [3,4]. While excessive Cys will lead to rheumatoid arthritis, Alzheimer’s disease, neural tube defects, Parkinson’s disease and cardiovascular disease [4]. Therefore, the accurate detection of Cys concentration is particularly important in health monitoring. However, Hcy and GSH in the blood have similar structures and reactive properties to Cys, which will interfere with the detection of Cys [5,6]. So, it is necessary to develop a real-time detection method for Cys with high sensitivity and selectivity to eliminate the impact of Hcy and GSH.

Fluorescent probes have the advantages of simple preparation, reliability, rapidity, low detection limit, multi-channel, high-throughput, low cost, small light damage, high sensitivity and real-time monitoring, and are widely used in the fields of biological detection and fluorescent imaging [2,7,8]. There are many reports in the literature that the detection range of Cys based on fluorescence sensor is µM, with a detection limit range of approximately 0.02–0.2 µM, and a Stokes shift of approximately 50–140 nm, which can be effectively applied to the detection of a low concentration of Cys [9,10,11,12,13,14,15,16,17,18]. Although many fluorescent probes have made a significant contribution to the detection of Cys, there are still many problems to be solved. For example, the emission of fluorescent probes can be adjusted, which makes the detection signal deeper into the target, especially for in vivo samples. Furthermore, a large Stokes shift and a low detection limit improve the sensitivity and stability of the fluorescent probe, which improves the sensitivity and stability of the fluorescent probe, and enables it to have a high detection accuracy and application range. In addition, Cys has similar structures and reactive properties with Hcy and GSH and coexists in the blood, which interferes with the detection of Cys. Therefore, it is necessary to develop a method to eliminate Hcy and GSH interference to monitor Cys in real time. At present, many fluorescent probes for Cys detection have been synthesized based on coumarin [1], naphthalene fluorescein [5], benzothiazole [19], cyanine [4], pyrene derivative [20], etc. Its mechanism includes the Michael addition, cyclization with aldehydes, disulfide bond cleavage reaction, and nucleophilic substitution [21,22]. However, few fluorescent probes have been reported to distinguish Cys, Hcy and GSH due to the similar reaction properties and structures of Cys, Hcy and GSH [1,5]. To prepare fluorescent probes with high sensitivity and good specificity for Cys, eliminating the interference of Hcy and GSH in Cys detection has become an important research direction for disease prevention. There are reports that the violet fluorescent probes have the characteristics of high energy, strong penetration and low light attenuation performance in the medium, which gives them the advantages of high sensitivity and strong anti-interference ability in biological detection [23,24]. Monovalent copper has the characteristics of good fluorescence performance and low cost, and the complexes formed with complexes containing N and S ligands have a high quantum yield and adjustable emission wavelength [25]. 2-mercaptobenzimidazole contains a mercapto group and has a strong ligand field [26], which was selected as the ligand for monovalent copper to form a fluorescence probe at the UV wavelength. Furthermore, the 2-mercaptobenzimidazole functionalized copper nanoparticles fluorescent probe is expected to eliminate the interference of Hcy and GSH. Cys has a stronger electronic supply capacity than Hcy and GSH, and is more likely to react with copper, resulting in fluorescence quenching of the probe [7].

In this study, 2-mercaptobenzimidazole-copper nanoparticles (MBI-CuNPs) fluorescent probes were constructed based on 2-mercaptobenzimidazole functionalized copper nanoparticles. The prepared MBI-CuNPs probe exhibits a strong fluorescence emission at 415 nm under the excitation of 200 nm. Furthermore, MBI-CuNPs fluorescent probes have a high response to Cys, which can eliminate the interference of Hcy and GSH. Moreover, MBI-CuNPs probes were successfully applied to the detection of Cys in serum, which has great advantages in abnormal Cys prevention and detection. 

## 2. Experiment

### 2.1. Chemical Reagents

Serum, sodium hydroxide (NaOH), sodium chloride (NaCl), lithium chloride (LiCl), ferric chloride (FeCl_3_), polyvinylpyrrolidone (PVP), 2-merhydryl benzimidazole (MBI) and cysteine (Cys) were obtained from Sangon Biotech (www.sangon.com). Potassium chloride (KCl), sodium borohydride (NaBH_4_), copper sulfate (CuSO_4_), calcium chloride (CaCl_2_), isoleucine (Iso), tryptophan (Trp), glycine (Gly), glutathione (GSH), ascorbic acid (Asc) and glucose (Glu) were obtained from Macklin (www.labgogo.com). Magnesium chloride (MgCl_2_), zinc chloride (ZnCl_2_), methionine (Met), lysine (Lys), threonine (Thr), Valine (Val) and homocysteine (Hcy) were purchased from Aladdin Reagent Co., Ltd. (Shanghai, China). The chemicals used in the experiment were analytically reagents.

### 2.2. Instrument

The fluorescence spectrum measurement was carried out on a fluorescence spectrophotometer (F-4600, Hitachi, Japan) with a working voltage of 650 V and an E_x_/E_m_ of 10.0/10.0 nm. The particle size of the copper nanoparticles was characterized by transmission electron microscopy (TEM) (TECNAI G2 F30 S-TWIN). The absorption spectrum was characterized by an ultraviolet-visible (UV-vis) spectrophotometer (LAMBDA 650, PerkinElmer). The valence state of the copper was characterized by X-ray photoelectron spectroscopy (XPS) (Thermo ESCALAB 250Xi). The functional groups of the products were characterized by Fourier transform infrared spectroscopy (FT-IR) (Bruker-V Vertex 70, Bruker, Karlsruhe). The composition of the product was characterized by X-ray diffraction (XRD) (BRUCKER D8 ADVANCE).

### 2.3. Preparation of MBI-CuNPs

**Figure 1:**
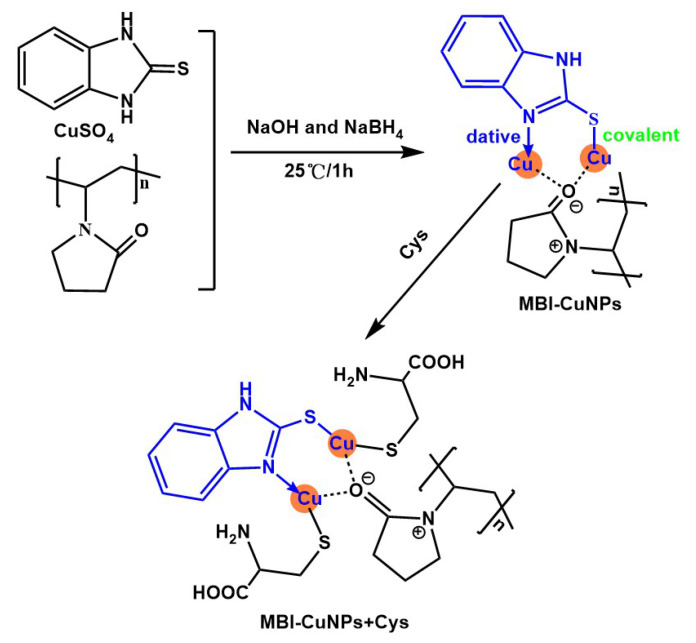
Synthesis of MBI-CuNPs and reaction of MBI-CuNPs with Cys.

Firstly, 64 mg (0.4 mM) CuSO_4_, 60 mg (0.4 mM) MBI and 44.4 mg (0.4 mM) PVP were dissolved into 80 mL deionized water and recorded as Sample 1. Secondly, 7.6 mg (0.2 mM) NaBH_4_ and 32 mg (0.8 mM) NaOH were dissolved into 10 mL deionized water and recorded as Sample 2. Finally, Sample 1 and Sample 2 were mixed and reacted at 25 ℃ for 1 h to obtain a blue solution, namely, the prepared 2-mercaptobenzimidazole-copper nanoparticles (MBI-CuNPs). Here, the MBI ligand was introduced to form a complex with the copper to prepare the copper nanoparticle fluorescent probes [26], and PVP was introduced to prevent the copper from being oxidized and improve the water solubility of the probes [27]. The synthetic scheme of the MBI-CuNPs probe is shown in Figure 1.

### 2.4. Detection of Cys

In the selective detection of Cys by MBI-CuNPs, each analyte solution with a concentration of 0.1 mM was prepared using deionized water. The analytes include NaCl, KCl, CaCl_2_, MgCl_2_, LiCl, ZnCl_2_, FeCl_3_, Thr, Gly, Cys, Lys, Trp, Met, Val, Iso, Asc, GSH, Hcy and Glu. Then, 0.5 mL of each analyte solution was added to 2 mL of MBI-CuNPs, respectively, and reacted for 2 min. Fluorescence determinations were performed under 200 nm light excitation at room temperature (25 °C). 

In the competitive detection of Cys by MBI-CuNPs, the coexistence solutions of Cys and each analyte were prepared using deionized water, respectively. The concentration of Cys in the coexistence solution was 0.1 mM, and the concentration of each analyte was also 0.1 mM. Analytes include NaCl, KCl, CaCl_2_, MgCl_2_, LiCl, ZnCl_2_, FeCl_3_, Thr, Gly, Lys, Trp, Met, Val, Iso, Asc, GSH, Hcy and Glu. Then, 0.5 mL of each coexisting solution was added to 2 mL of MBI-CuNPs, respectively, for 2 min, and the fluorescence was determined under 200 nm light excitation at room temperature (25 °C).

In the quantitative detection of Cys by MBI-CuNPs, the Cys solutions of different concentrations were prepared in deionized water, and then 0.5 mL of each Cys solution was added to 2 mL of MBI-CuNPs for 2 min, respectively. Its fluorescence was determined under 200 nm light excitation at room temperature (25 °C). 

### 2.5. Detection of Cys in Serum

Firstly, the serum (supernatant of hybridoma cell culture secreting monoclonal antibodies against human type A antigen) was diluted 10 times with deionized water, and serum samples containing different concentrations of Cys were prepared by the standard addition method. The concentrations of Cys in each serum sample were 0, 5, 10, 20, 30, 40, 50 and 65 µM, respectively. In this experiment, the purchased serum had a large turbidity and needed to be diluted to ensure the accuracy of the test. Secondly, serum samples containing 65 µM of all analytes were prepared, including NaCl, KCl, CaCl_2_, MgCl_2_, LiCl, ZnCl_2_, FeCl_3_, Thr, Gly, Cys, Lys, Trp, Met, Val, Iso, Asc, GSH, Hcy and Glu. Finally, 0.5 mL of each sample was added to 2 mL of MBI-CuNPs, respectively, and reacted for 2 min. Its fluorescence was determined under 200 nm light excitation at room temperature (25 °C). 

## 3. Results and Discussion

### 3.1. Characterization of MBI-CuNPs

**Figure 2:**
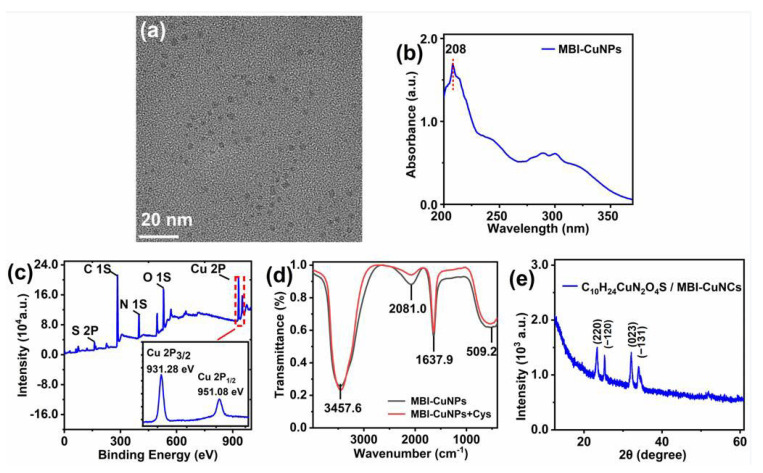
(**a**) TEM images of MBI-CuNPs. (**b**) Absorption spectra of MBI-CuNPs. (**c**) XPS analysis of MBI-CuNPs. Inset: XPS analysis of copper. (**d**) FT-IR spectra study of MBI-CuNPs and MBI-CuNPs+Cys. (**e**) XRD patterns of MBI-CuNPs.

The TEM of Figure 2a shows that the prepared MBI-CuNPs have a regular shape, uniform distribution, and a particle size of about 1.7 nm. In Figure 2b, the UV-Vis absorption peak of the MBI-CuNPs is located at 208 nm, indicating that there is no large aggregation of CuNPs because the absorption peak of CuNPs is related to the particle size of CuNPs [28,29,30]. Figure 2c shows the elemental composition of the MBI-CuNPs, in which S, C and N are provided by MBI and Cu is provided by CuSO_4_ in the MBI-CuNPs, while the O element comes from the PVP adsorbed on the CuNPs. In the inset of Figure 2c, the peaks of Cu 2P_3/2_ and Cu 2P_1/2_ are located at 931.28 eV and 951.08 eV, respectively, with an energy gap of 19.80 eV, which shows that CuNPs are composed of Cu(I) [30,31]. In Figure 2d, the absorption peaks at 3457.6, 2080.0 and 1637.9 cm^−1^ are the vibrational of the O–H bond in the water molecule adsorbed on the PVP, the C-H bond on the benzene ring of MBI, and the C=O double bond on the PVP, respectively [32,33,34]. The absorption band at 509.2 cm^−1^ corresponds to the vibrational absorption of the S–Cu bond and N–Cu bond formed by MBI and Cu(I) [35]. Compared with the FT-IR spectra of MBI-CuNPs, the vibration of MBI-CuNPs+Cys decreases at 2081.0 cm^−1^, which may be due to the electrostatic interaction between Cys and MBI weakening the vibration of the C–H bond on the benzene ring of the MBI. In addition, the peak strength of MBI-CuNPs+Cys at 509.2 cm^−1^ remains basically unchanged, which indicates that the S–Cu and N–Cu bonds still exist after adding Cys to the MBI-CuNPs. The XRD of the MBI-CuNPs are shown in Figure 2e. The diffraction peaks at 23.3°, 25.2°, 32.1° and 33.9° are (220), (−120), (023) and (−131) crystal planes of ethylamine butylcopper (C_10_H_24_CuN_2_OS) (JCPDS, 31-1626), respectively, which is consistent with the molecular formula C_7_H_6_N_2_S of MBI, while Cu comes from the CuNPs, and the excess C, H, and O elements may come from the PVP. It shows that MBI is coordinated with monovalent copper, which is consistent with the FT-IR determination results of the MBI-CuNPs in Figure 2d.

**Figure 3:**
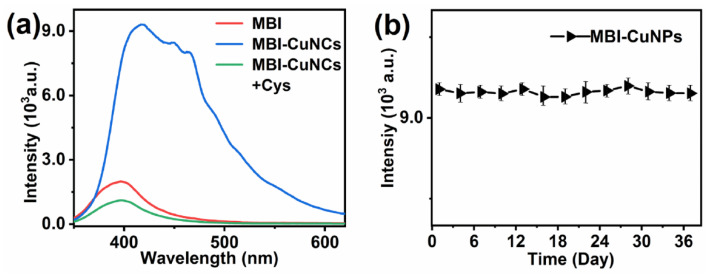
(**a**) Fluorescence spectra of MBI, MBI-CuNPs and MBI-CuNPs+Cys. (**b**) Fluorescence changes of MBI-CuNPs probe in 37 days.

The fluorescence of MBI, MBI-CuNPs and MBI-CuNPs+Cys were measured under 200 nm excitation at room temperature (25 °C). Figure 3a shows that MBI only exhibits weak fluorescence at 395 nm, while the MBI-CuNPs probes exhibit strong fluorescence emission at 415 nm. This may be due to the formation of the charge transfer transition between MBI and monovalent copper in the MBI-CuNPs, thus improving the fluorescence quantum yield of the MBI-CuNPs. In addition, the fluorescence of the MBI-CuNPs was significantly quenched after reacting with 65 µM Cys for 2 min, which could be used for sensitive and rapid detection of Cys. This is attributed to the fact that the fluorescence peak of the MBI-CuNPs probe is located in the violet region. It has the characteristics of high energy, strong penetration and low light decay performance in solution, which makes the MBI-CuNPs probe show as being highly sensitive to Cys [23,24]. Figure 3b shows that the fluorescence properties of the MBI-CuNPs probe are stable within 37 days and can be used for practical detection. 

### 3.2. Response of MBI-CuNPs Probe to Cys

To study the specificity of the MBI-CuNPs probe to Cys, the fluorescence responses of MBI-CuNPs probes to different analytes were studied. The serum contains Na^+^, K^+^, Li^+^, Ca^2+^, Mg^2+^, Zn^2+^, Fe^3+^, Gly, Lys, Try, Met, Iso, Thr, GSH, Val, Arg, Tyr, Glu and VC, as well as the aforementioned components. So, we must test whether the probe responds to the above ions and components in specific experiments.

**Figure 4:**
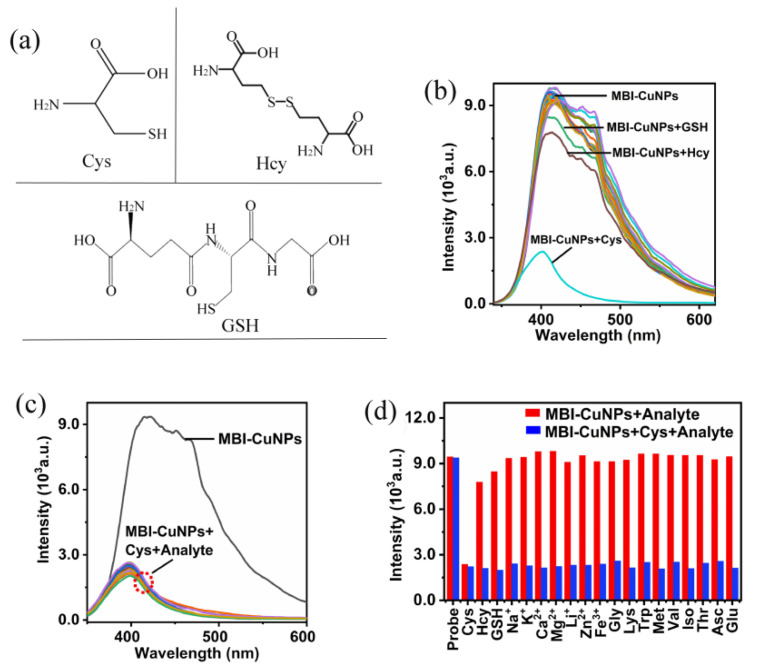
(**a**) The structure diagram of Cys, Hcy and GSH. (**b**) Selective detection of Cys by the MBI-CuNPs. (**c**) Competitive detection of Cys by MBI-CuNPs probe. (**d**) Fluorescence intensity at 415 nm for each spectrum in (**b**,**c**).

Figure 4a is the structure diagram of the Cys, Hcy and GSH, in which it can be seen that they have similar structures. However, the zero position of the sulfhydryl (-SH) group on Cys contains an amino (-NH_2_) group, which has a stronger electron-donating capacity than Hcy and GSH. The fluorescence response of the MBI-CuNPs probe to each analyte is reflected in Figure 4b. The fluorescence of MBI-CuNPs was significantly quenched by Cys, the fluorescence of the MBI-CuNPs probe was quenched by GSH to a lesser extent and the fluorescence of the MBI-CuNPs probe quenched by Hcy is slightly greater than GSH. This may be due to the fact that the ortho position of the thiol group on Cys contains the amino group, which has a stronger electron supply ability than Hcy and GSH and is more likely to cause the fluorescence quenching of the MBI-CuNPs probe. In addition, other analytes have little effect on the fluorescence of MBI-CuNPs. To further explain the anti-interference ability of MBI-CuNPs probes in Cys detection, the fluorescence response of MBI-CuNPs to each coexistence was studied. As shown in Figure 4c, the results show that other analytes do not interfere with the reaction between MBI-CuNPs and Cys, indicating that the MBI-CuNPs probes have a strong anti-interference ability in the detection of Cys and can selectively detect Cys. The fluorescence intensity at 415 nm in Figure 4a,c is visually reflected in Figure 4d. Figure 4d also clearly shows that the influence of other analytes on the detection of Cys can be negligible, except that Hcy and GSH have little interference in the detection of Cys. The above analysis results show that MBI-CuNPs probes can be used for the selective detection of Cys.

**Figure 5:**
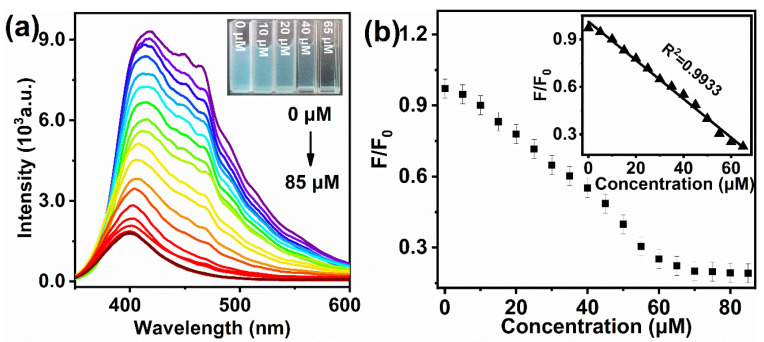
(**a**) Fluorescence responses of MBI-CuNPs probe to different concentrations of Cys. (**b**) Linear relationship between fluorescence of MBI-CuNPs probe and Cys concentration.

The fluorescence responses of MBI-CuNPs probes to Cys (0–85 µM) were studied, and the Cys concentrations were 0, 0.05, 5, 10, 15, 20, 25, 30, 35, 40, 45, 50, 55, 60, 65, 70, 75, 80 and 85 µM, respectively. As shown in Figure 5a, the MBI-CuNPs probes are highly fluorescence dependent on Cys, and the inset shows that the color of the solution disappears significantly after the reaction between MBI-CuNPs and Cys, indicating that MBI-CuNPs have an obvious fading reaction with Cys. Figure 5b shows that the fluorescence quenching rate F/F_0_ of the MBI-CuNPs probe has a good linear relationship with the Cys concentration Q of 0.05–65 µM. The linear fitting relationship is F/F_0_ = −0.0122 Q + 1.0124, and the correlation coefficient R^2^ = 0.9933. The detection limit (LOD) (LOD = 3δ/k, *n* = 10) of MBI-CuNPs for Cys is 52 nM.

In Table 1, the probe prepared in this study is compared with the probe currently reported for the detection of Cys, and it was found that the MBI-CuNPs probe showed a wide linear range and a low detection limit.

### 3.3. Analysis of Cys in Serum Samples

The MBI-CuNPs probe is expected to be used for serum analysis to demonstrate the feasibility of the MBI-CuNPs probe in the analysis of Cys in serum. Blank serum samples were determined using the MBI-CuNPs probe, and the fluorescence intensity F/F_0_ was replaced into the fitting relationship F/F_0_ = −0.0125Q + 1.0263 to obtain the concentration Q_0_ of Cys in serum as 3.3 µM. In addition, the serum samples containing different concentrations of Cys were further detected by the MBI-CuNPs probe, and the detection concentration Q_d_ of Cys was calculated by substituting the measured fluorescence intensity F/F_0_ into the fitting relationship F/F_0_ = −0.0125Q + 1.0263. The standard addition concentrations of Cys in serum samples were 5, 10, 20, 30, 40, 50 and 65 µM, respectively. In order to evaluate the closeness of the measurement results to the true value, the absolute recovery rate (Q_d_ − Q_0_)/Q of Cys was calculated, where Q is the standard added concentration of Cys in the serum samples [35,36]. The absolute recovery rate and relative standard deviation were 90.23–97.00% and 0.41–2.73, respectively. Meanwhile, serum samples containing 65 µM of all analytes were also detected, and the absolute recovery rate and relative standard deviation were 93.80% and 1.96, respectively. The measured data are shown in Table 2, which indicated that the MBI-CuNPs probe could be used for the actual detection of cysteine in serum.

### 3.4. Research on Response Mechanism

**Figure 6:**
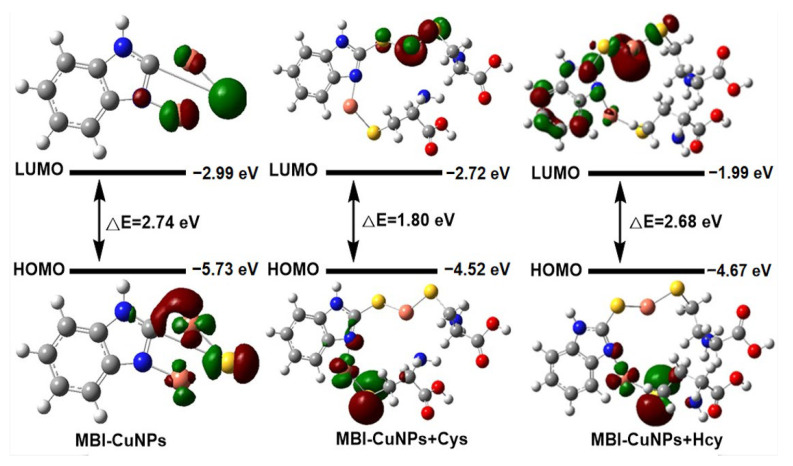
Frontier molecular orbital of MBI-CuNPs, MBI-CuNPs+Cys and MBI-CuNPs+Hcy.

According to the characterization results, the response mechanism of the MBI-CuNPs probe to Cys was analyzed. As shown in Figure 1, the sulfhydryl group of Cys will coordinate with copper in MBI-CuNPs through the S–Cu bond after adding Cys to the MBI-CuNPs solution, thus quenching the fluorescence of MBI-CuNPs. In Figure 6, to further analyze the fluorescence quenching mechanism of Cys on MBI-CuNPs, the frontier molecular orbitals of MBI-CuNPs, MBI-CuNPs+Cys and MBI-CuNPs+Hcy were calculated by Gaussian 09W Opt+Freq Ground State DFT. The highest occupied molecular orbital (HOMO) of MBI-CuNPs is mainly located on the monovalent copper, partially on S, while the lowest unoccupied molecular orbital (LUMO) is mainly located on S, partially on monovalent copper. Therefore, the metal–ligand charge transfer (MLCT) transition will occur in MBI-CuNPs under light excitation, resulting in fluorescence. 

In this paper, we calculate the emission peak through the band gap using the formula λ = hc/E. The energies of HOMO and LUMO for MBI-CuNPs are −5.73 eV and −2.99 eV, respectively, with a bandgap of 2.74 eV, corresponding to the emission peak of 452 nm, which is close to the emission peak of MBI-CuNPs at 415 nm. In this part, we can find that the calculated bandgap width is smaller than that measured in the experiment because the influence of PVP adsorption on the surface of the nanoparticles is not considered in the calculation. The HOMO of MBI-CuNPs+Cys is located in the skeleton connected with N on the MBI, and the LUMO is located in the skeleton connected with S on the MBI. Therefore, the transition from HOMO to LUMO in MBI-CuNPs+Cys needs to span the MBI, so it is difficult to occur, leading to the fluorescence quenching of MBI-CuNPs. The HOMO of MBI-CuNPs+Hcy is located in the skeleton connected with N on the MBI, and the LUMO is located in the skeleton connected with S on the MBI and on the MBI. Therefore, the charge transfer transition from HOMO to LUMO on MBI can still occur in MBI-CuNPs+Hcy under light excitation, but the transition is weakened, resulting in a smaller degree of fluorescence quenching of MBI-CuNPs. This is because the zero position of the thiol group on Hcy does not contain amino groups, and its electron supply ability is weaker than Cys [37]. In addition, the energies of HOMO and LUMO for MBI-CuNPs+Hcy are −4.67 eV and −1.99 eV, respectively, with a bandgap of 2.68 eV, which corresponds to the emission peak of 462 nm, and it is close to the emission peak of MBI-CuNPs+Hcy at 413 nm. Compared with the frontier molecular orbital of MBI-CuNPs, MBI-CuNPs+Cys and MBI-CuNPs+Hcy have higher HOMO energies of −4.52 eV and −4.67 eV, respectively, which is attributed to the fact that Cys and Hcy contain the electron donor group thiol. The thiol can coordinate with monovalent copper in MBI-CuNPs through the Cu–S bond and increase the HOMO energy of MBI-CuNPs [38]. In addition, the HOMO energy of MBI-CuNPs+Cys is higher than that of MBI-CuNPs+Hcy, which makes it easier to quench the fluorescence of MBI-CuNPs. As reported in the literature, Cys enables metal complexes with higher excitation orbital energy than Hcy, which makes it easier to cause fluorescence quenching of complexes [38]. 

## 4. Conclusions

In this study, MBI-CuNPs were constructed based on MBI and monovalent copper. MBI-CuNPs have strong fluorescence emission at 415 nm under the excitation of 200 nm at room temperature, and the fluorescence intensity can be quenched by Cys for effective detection of the concentration of Cys. Furthermore, the MBI-CuNPs fluorescent probe can eliminate the interference of Hcy and GSH in selective detection, which was attributed to the strong electron-supplying ability of Cys making it easier to quench the fluorescence of MBI-CuNPs fluorescent probes. Experiments show that the linear range and detection limit of the MBI-CuNPs probe for Cys are 0.05–65 µM and 52 nM, respectively. In the real serum detection of the MBI-CuNPs probe, the absolute recovery rate of Cys reached 90.23–97.00%. This high sensitivity and excellent selectivity of the fluorescent probe is expected to have great potential in abnormal Cys prevention and detection. 

## Figures and Tables

**Table 1:** The performance of this probe is compared with the recently reported probe in detecting Cys.

Probe	λ_ex_/nm	λ_em_/nm	LOD/µM	Linear Range/μM	Literature
HDFC	390	500	0.19	10–70	[1]
BC	400	452	0.366	0–100	[2]
Cy-NB	560	640	0.2	35–100	[3]
Probe 1	612	690	0.18	0–25	[4]
NR-NBD	670	716	0.027	0.5–25	[6]
NN	510	559	0.05	-	[7]
YF	418	496	2.5	0–30	[19]
NBD-O-1	-	550	0.061	0–27	[20]
MBI-CuNPs	200	415	0.052	0.05–65	This work

**Table 2:** The feasibility of using MBI-CuNPs probe to detect Cys in real serum samples was evaluated.

Sample	Cys (µM)	Fund Cys (µM)	Absolute Recovery Rate (%)	RSD (%)
Serum sample	0	3.30	-	-
	5	8.15	97.00	0.41
	10	12.60	93.07	0.97
	20	21.34	90.23	0.49
	30	32.04	95.83	0.99
	40	39.97	91.69	2.73
	50	48.89	91.19	1.56
	65	63.76	93.02	1.86
	65 ^a^	64.28 ^a^	93.80 ^a^	1.96 ^a^

^a^: Serum samples containing 65 µM of NaCl, KCl, CaCl_2_, MgCl_2_, LiCl, ZnCl_2_, FeCl_3_, Thr, Gly, Cys, Lys, Trp, Met, Val, Iso, Asc, GSH, Hcy and Glu.

## Data Availability

Data available in a publicly accessible repository.
